# Supplementary material for: Unveiling the Bioactive Potential of the Invasive Jellyfish Phyllorhiza punctata Through Integrative Transcriptomic and Proteomic Analyses
Source: Biomolecules. 2025 Aug 4;15(8):1121. doi: 10.3390/biom15081121 (PMC12383608; doi:10.3390/biom15081121)
Supplement: Supplementary file 1 [file biomolecules-15-01121-s001.zip › Suplementary Material/FileS3/PEPFOLD4/XP_065067012_11913/clusters.html]

 PEP-FOLD clusters


Download archive of all models  here   

|  |  |  |
| --- | --- | --- |
| # | file | sOPEP |
| # cluster 1 - Size: 27 |  |  |
|  | XP\_065067012\_11913-model1 | -23.6812 |
|  | XP\_065067012\_11913-model1.1 | -23.6771 |
|  | XP\_065067012\_11913-model1.2 | -23.6771 |
|  | XP\_065067012\_11913-model1.3 | -23.6462 |
|  | XP\_065067012\_11913-model1.4 | -23.5207 |
|  | XP\_065067012\_11913-model1.5 | -23.519 |
|  | XP\_065067012\_11913-model1.6 | -23.5168 |
|  | XP\_065067012\_11913-model1.7 | -23.4833 |
|  | XP\_065067012\_11913-model1.8 | -23.4586 |
|  | XP\_065067012\_11913-model1.9 | -23.4197 |
|  | XP\_065067012\_11913-model1.10 | -23.3538 |
|  | XP\_065067012\_11913-model1.11 | -23.3266 |
|  | XP\_065067012\_11913-model1.12 | -23.2666 |
|  | XP\_065067012\_11913-model1.13 | -23.2458 |
|  | XP\_065067012\_11913-model1.14 | -23.2392 |
|  | XP\_065067012\_11913-model1.15 | -23.2124 |
|  | XP\_065067012\_11913-model1.16 | -23.1787 |
|  | XP\_065067012\_11913-model1.17 | -23.154 |
|  | XP\_065067012\_11913-model1.18 | -23.1308 |
|  | XP\_065067012\_11913-model1.19 | -23.0112 |
|  | XP\_065067012\_11913-model1.20 | -22.9326 |
|  | XP\_065067012\_11913-model1.21 | -22.8839 |
|  | XP\_065067012\_11913-model1.22 | -22.8388 |
|  | XP\_065067012\_11913-model1.23 | -22.7184 |
|  | XP\_065067012\_11913-model1.24 | -22.6927 |
|  | XP\_065067012\_11913-model1.25 | -22.654 |
|  | XP\_065067012\_11913-model1.26 | -22.5749 |
| # cluster 2 - Size: 66 |  |  |
|  | XP\_065067012\_11913-model2 | -23.438 |
|  | XP\_065067012\_11913-model2.1 | -23.3038 |
|  | XP\_065067012\_11913-model2.2 | -23.2991 |
|  | XP\_065067012\_11913-model2.3 | -23.2945 |
|  | XP\_065067012\_11913-model2.4 | -23.1928 |
|  | XP\_065067012\_11913-model2.5 | -23.1461 |
|  | XP\_065067012\_11913-model2.6 | -23.0661 |
|  | XP\_065067012\_11913-model2.7 | -23.0539 |
|  | XP\_065067012\_11913-model2.8 | -23.0324 |
|  | XP\_065067012\_11913-model2.9 | -22.9778 |
|  | XP\_065067012\_11913-model2.10 | -22.9693 |
|  | XP\_065067012\_11913-model2.11 | -22.9361 |
|  | XP\_065067012\_11913-model2.12 | -22.9271 |
|  | XP\_065067012\_11913-model2.13 | -22.9003 |
|  | XP\_065067012\_11913-model2.14 | -22.8774 |
|  | XP\_065067012\_11913-model2.15 | -22.8615 |
|  | XP\_065067012\_11913-model2.16 | -22.8535 |
|  | XP\_065067012\_11913-model2.17 | -22.8528 |
|  | XP\_065067012\_11913-model2.18 | -22.8302 |
|  | XP\_065067012\_11913-model2.19 | -22.8255 |
|  | XP\_065067012\_11913-model2.20 | -22.8192 |
|  | XP\_065067012\_11913-model2.21 | -22.8021 |
|  | XP\_065067012\_11913-model2.22 | -22.7963 |
|  | XP\_065067012\_11913-model2.23 | -22.7963 |
|  | XP\_065067012\_11913-model2.24 | -22.785 |
|  | XP\_065067012\_11913-model2.25 | -22.785 |
|  | XP\_065067012\_11913-model2.26 | -22.7837 |
|  | XP\_065067012\_11913-model2.27 | -22.7748 |
|  | XP\_065067012\_11913-model2.28 | -22.754 |
|  | XP\_065067012\_11913-model2.29 | -22.7096 |
|  | XP\_065067012\_11913-model2.30 | -22.6997 |
|  | XP\_065067012\_11913-model2.31 | -22.6917 |
|  | XP\_065067012\_11913-model2.32 | -22.6908 |
|  | XP\_065067012\_11913-model2.33 | -22.6626 |
|  | XP\_065067012\_11913-model2.34 | -22.6179 |
|  | XP\_065067012\_11913-model2.35 | -22.6179 |
|  | XP\_065067012\_11913-model2.36 | -22.5762 |
|  | XP\_065067012\_11913-model2.37 | -22.5655 |
|  | XP\_065067012\_11913-model2.38 | -22.5587 |
|  | XP\_065067012\_11913-model2.39 | -22.5505 |
|  | XP\_065067012\_11913-model2.40 | -22.5432 |
|  | XP\_065067012\_11913-model2.41 | -22.534 |
|  | XP\_065067012\_11913-model2.42 | -22.5308 |
|  | XP\_065067012\_11913-model2.43 | -22.5204 |
|  | XP\_065067012\_11913-model2.44 | -22.5118 |
|  | XP\_065067012\_11913-model2.45 | -22.5082 |
|  | XP\_065067012\_11913-model2.46 | -22.5058 |
|  | XP\_065067012\_11913-model2.47 | -22.5013 |
|  | XP\_065067012\_11913-model2.48 | -22.4893 |
|  | XP\_065067012\_11913-model2.49 | -22.4777 |
|  | XP\_065067012\_11913-model2.50 | -22.477 |
|  | XP\_065067012\_11913-model2.51 | -22.4435 |
|  | XP\_065067012\_11913-model2.52 | -22.4034 |
|  | XP\_065067012\_11913-model2.53 | -22.4029 |
|  | XP\_065067012\_11913-model2.54 | -22.3931 |
|  | XP\_065067012\_11913-model2.55 | -22.3904 |
|  | XP\_065067012\_11913-model2.56 | -22.3813 |
|  | XP\_065067012\_11913-model2.57 | -22.3781 |
|  | XP\_065067012\_11913-model2.58 | -22.3523 |
|  | XP\_065067012\_11913-model2.59 | -22.3496 |
|  | XP\_065067012\_11913-model2.60 | -22.3459 |
|  | XP\_065067012\_11913-model2.61 | -22.3209 |
|  | XP\_065067012\_11913-model2.62 | -22.2832 |
|  | XP\_065067012\_11913-model2.63 | -22.2787 |
|  | XP\_065067012\_11913-model2.64 | -22.2746 |
|  | XP\_065067012\_11913-model2.65 | -22.2325 |
| # cluster 3 - Size: 7 |  |  |
|  | XP\_065067012\_11913-model3 | -22.841 |
|  | XP\_065067012\_11913-model3.1 | -22.8404 |
|  | XP\_065067012\_11913-model3.2 | -22.8332 |
|  | XP\_065067012\_11913-model3.3 | -22.6996 |
|  | XP\_065067012\_11913-model3.4 | -22.3057 |
|  | XP\_065067012\_11913-model3.5 | -22.2947 |
|  | XP\_065067012\_11913-model3.6 | -22.2913 |
|  |  |  |
